# Supplementary material for: Research on the evaluation of China’s Supply Chain Finance policy based on text mining
Source: PLoS One. 2025 Mar 10;20(3):e0317743. doi: 10.1371/journal.pone.0317743 (PMC11893131; doi:10.1371/journal.pone.0317743)
Supplement: Appendix — (DOCX) [file pone.0317743.s001.docx]

**Appendix. Table of variable assignments for representative SCF policy samples**

| **Primary Variables** | **Secondary Variables** | **P1** | **P2** | **P3** | **P4** | **P5** | **P6** | **P7** | **P8** | **P9** |
| --- | --- | --- | --- | --- | --- | --- | --- | --- | --- | --- |
| X_1_ | X_1-1_ | 1 | 0 | 1 | 1 | 1 | 1 | 1 | 1 | 1 |
|  | X_1-2_ | 0 | 0 | 1 | 1 | 0 | 0 | 1 | 0 | 1 |
|  | X_1-3_ | 1 | 1 | 1 | 1 | 1 | 1 | 1 | 0 | 1 |
|  | X_1-4_ | 0 | 1 | 1 | 0 | 1 | 1 | 1 | 1 | 1 |
| X_2_ | X_2-1_ | 0 | 0 | 0 | 0 | 1 | 1 | 1 | 1 | 1 |
|  | X_2-2_ | 1 | 1 | 1 | 1 | 0 | 0 | 0 | 0 | 0 |
| X_3_ | X_3-1_ | 1 | 1 | 1 | 0 | 0 | 0 | 0 | 0 | 1 |
|  | X_3-2_ | 0 | 0 | 0 | 1 | 1 | 1 | 1 | 1 | 0 |
| X_4_ | X_4-1_ | 1 | 0 | 0 | 1 | 0 | 1 | 1 | 0 | 0 |
|  | X_4-2_ | 1 | 1 | 0 | 1 | 0 | 0 | 0 | 0 | 0 |
|  | X_4-3_ | 0 | 0 | 1 | 1 | 0 | 0 | 1 | 0 | 1 |
|  | X_4-4_ | 0 | 0 | 0 | 1 | 1 | 1 | 1 | 1 | 1 |
|  | X_4-5_ | 1 | 1 | 1 | 1 | 1 | 1 | 1 | 1 | 1 |
| X_5_ | X_5-1_ | 0 | 0 | 0 | 0 | 0 | 0 | 1 | 0 | 0 |
|  | X_5-2_ | 0 | 0 | 0 | 0 | 0 | 0 | 0 | 0 | 0 |
|  | X_5-3_ | 1 | 0 | 1 | 0 | 0 | 0 | 1 | 1 | 1 |
|  | X_5-4_ | 0 | 0 | 0 | 0 | 0 | 0 | 0 | 0 | 0 |
|  | X_5-5_ | 0 | 0 | 1 | 1 | 0 | 0 | 0 | 0 | 0 |
|  | X_5-6_ | 0 | 0 | 1 | 1 | 0 | 0 | 1 | 1 | 1 |
|  | X_5-7_ | 0 | 0 | 1 | 1 | 0 | 1 | 1 | 1 | 1 |
|  | X_5-8_ | 0 | 0 | 1 | 0 | 0 | 0 | 1 | 0 | 1 |
|  | X_5-9_ | 0 | 1 | 1 | 0 | 1 | 0 | 0 | 0 | 0 |
|  | X_5-10_ | 0 | 0 | 0 | 0 | 0 | 0 | 0 | 1 | 0 |
|  | X_5-11_ | 1 | 1 | 0 | 0 | 1 | 1 | 0 | 0 | 1 |
| X_6_ | X_6-1_ | 1 | 0 | 1 | 1 | 1 | 1 | 1 | 1 | 1 |
|  | X_6-2_ | 1 | 1 | 1 | 0 | 0 | 0 | 1 | 1 | 0 |
|  | X_6-3_ | 1 | 1 | 1 | 0 | 0 | 0 | 1 | 1 | 1 |
|  | X_6-4_ | 0 | 0 | 1 | 1 | 0 | 1 | 1 | 1 | 1 |
| X_7_ | X_7-1_ | 1 | 1 | 1 | 0 | 1 | 1 | 0 | 0 | 1 |
|  | X_7-2_ | 0 | 0 | 0 | 1 | 1 | 1 | 1 | 1 | 1 |
|  | X_7-3_ | 0 | 0 | 1 | 0 | 0 | 0 | 1 | 0 | 1 |
| X_8_ | X_8-1_ | 0 | 1 | 1 | 0 | 0 | 1 | 1 | 1 | 0 |
|  | X_8-2_ | 1 | 1 | 1 | 1 | 1 | 0 | 1 | 1 | 1 |
|  | X_8-3_ | 1 | 1 | 1 | 1 | 0 | 1 | 1 | 1 | 1 |
| X_9_ | X_9-1_ | 1 | 0 | 1 | 1 | 1 | 1 | 1 | 0 | 1 |
|  | X_9-2_ | 0 | 0 | 0 | 0 | 0 | 0 | 0 | 1 | 0 |
|  | X_9-3_ | 0 | 1 | 0 | 0 | 0 | 0 | 0 | 0 | 0 |
| X_10_ | X_10-1_ | 0 | 0 | 0 | 0 | 0 | 0 | 0 | 0 | 0 |
|  | X_10-2_ | 1 | 0 | 0 | 1 | 0 | 0 | 0 | 1 | 0 |
|  | X_10-3_ | 0 | 0 | 0 | 0 | 0 | 0 | 0 | 0 | 0 |
|  | X_10-4_ | 0 | 1 | 1 | 0 | 1 | 1 | 1 | 0 | 1 |
