# Supplementary material for: Research on the evaluation of China’s Supply Chain Finance policy based on text mining
Source: PLoS One. 2025 Mar 10;20(3):e0317743. doi: 10.1371/journal.pone.0317743 (PMC11893131; doi:10.1371/journal.pone.0317743)
Supplement: Attachment — Submitted filename: Response to Reviewers.docx [file pone.0317743.s003.docx]

Dear Editor and Reviewers,

Thank you for your letter and for there viewers' comments concerning our manuscript entitled "Research on the Evaluation of China's SCF Policy Based on Text Mining" (ID: PONE-D-24-45592). Those comments are all valuable and very helpful for revising and improving our paper, as well as the important guiding significance to our research. We have studied comments carefully and have made corrections, which we hope meet with approval. Revised portions are marked in red underline in the paper.

The main corrections in the paper and the responses to the reviewer's comments are as flowing:

**Responds to journal requirements:**

1. **Comment:**

Please ensure that your manuscript meets PLOS ONE's style requirements, including those for file naming.

**Response:**

We apologize that our previous submission did not fully meet your journal's requirements. At present, we have carefully modified the article format according to the style template file you provided.

2. **Comment:**

Please ensure that you include a title page within your main document.

**Response:**

The revised manuscript has placed the title page at the beginning of the manuscript file.

3. **Comment:**

Please state what role the funders took in the study.

**Response:**

The second author's responsibilities for this study are: Conceptualization, Supervision, and Validation. In addition, after our confirmation, this study did not obtain any funding support, so we will modify the corresponding information in the revised submission.

4. **Comment:**

Recommend all authors decide on a data sharing plan before acceptance.

**Response:**

After discussion among all authors, we confirm acceptance of your journal's data sharing plan: all data will be freely accessible after the manuscript is accepted for publication. But we don't know what actions we should take to show that we support the plan.

5. **Comment:**

Please ensure that you include a title page within your main document.

**Response:**

The revised manuscript now lists all authors and all affiliations in accordance with your journal's instructions for authors and clearly indicates the corresponding author.

6. **Comment:**

Please review your reference list to ensure that it is complete and correct.

**Response:**

Thank you for your reminder. We have added 36 new references in the revised paper. In addition, we have carefully checked all the cited literature to ensure that there are no studies with "retracted" status among them.

At the end of this letter we provide detailed information on the changes to the references.

**Responds to the reviewers' comments:**

**Reviewer #1:**

1. **Comment:**

It is recommended to avoid using abbreviations in the title, such as "SCF" Writing out "Supply Chain Finance" will help readers quickly understand the subject of the study.

**Response:**

Your suggestion is very accurate and helpful, and we have changed the "SCF" in the title to "Supply Chain Finance".

2. **Comment:**

Consider expanding the abstract to more comprehensively reflect the core elements of the study, including the research purpose, methodology, findings, and conclusions. Including specific information, such as sample size, would further enrich the abstract and draw readers' interest.

**Response:**

According to your suggestion, we have revised the abstract. Specifically, we have adopted a more coherent writing logic and enriched the content of the research purpose and methodology with specific research information. You can see them in lines 21 to 29 of the revised manuscript.

3. **Comment:**

The methodology-related content currently included in the "Literature Review" section should be moved to the "Research Methodology" section to improve logical flow. Additionally, expanding the literature review to include a broader discussion of SCF policies, the BERTopic model, and the PMC index model will provide a stronger theoretical foundation for the analysis. To strengthen the literature review, consider adding more studies related to SCF policy development and theoretical background, particularly regarding the applications of the BERTopic model in policy analysis and the theoretical basis for using the PMC index model. This will provide stronger support for the research design and methodology choices.

**Response:**

According to your requirements and suggestions, we have made the following changes:

First, we adjusted the position and expression strategy of the contents related to computer technology in the literature review. In the revised manuscript, they are located in lines 209-229 as the "Main technologies for indicator selection" section. This section is used to explain how to achieve the technical requirements of this study, that is, to build a more optimized policy evaluation index system, the development trajectory of related technologies and their advantages and disadvantages, and why this study chooses to use the BERTopic model to optimize the construction of the index system.

Second, we revised the literature review section. Specifically, we added a review of SCF policy-related research (lines 85-105) and revised the review of policy evaluation methods (lines 134-172).In the "SCF Policy-Related Research" section, we analyzed the main directions and shortcomings of existing research, and introduced a broader discussion on combining technical means with SCF policy evaluation. In the "Policy Evaluation Methods" section, we systematically sorted out the existing policy evaluation methods, and introduced the PMC index model by comparing the evaluation performance of different models. Furthermore, based on the subjective problem of the PMC index model, we introduced the idea of using technical means to optimize the process of selecting policy evaluation indicators.

Thank you for your careful reading of our study and your professional and constructive comments. We believe that the revisions in this section more effectively support the research design and method selection.

4. **Comment:**

Certain sections could be expressed more concisely to improve readability. Consider revising sentence structure to enhance the overall quality of English expression.

**Response:**

According to your request, we have rechecked the academic norms of each paragraph and tried to present it in a more readable language. Specifically, the lines we have revised are as follows: 37-83, 245-255, 257-261, 271-274, 283-314, 335-349, 357-364.

**Reviewer #2:**

1. **Comment:**

Under the part of literature you need to define your technical terms like, text mining, supply chain finance, and cluster analysis so that the readers can clearly understand the context of your study.

**Response:**

Thank you for your sincere comments. We have added a "Concept definition" section between "Literature Review" and "Research Framework" (lines 173-178). This section introduces the definitions of the main terms used in the article and how they are interpreted in this study. In addition, we have plotted the results in a table to improve readability.

**Reviewer #3:**

Thank you for taking the time to read our research, and we're glad it received such high praise from you. We wish you all the best!

**Reviewer #4:**

1. **Comment:**

Ethical approval seems missing, If authors can provide the details in methodology, it will be much better.

**Response:**

Thank you for taking the time to read our research. The questions you raised are very important.

First of all, we attach great importance to the research norms regarding ethics. However, in this study, no living organisms were involved in the experiments, and all the data or texts used as research subjects can be publicly queried on the Internet. Therefore, we did not add the ethical approval section to the paper. Of course, if you think it is necessary to include this statement, we will consider it.

Second, regarding the details of the methodology, in the revised paper, we added "Main technologies for indicator selection" (lines 209-229), which, combined with the "Policy Evaluation Methods" in the literature review, will help readers better understand the methodology used in this study.

**Reference revision list:**

Due to the revision of some parts of the literature review, the references cited in the corresponding parts need to be reconsidered. Specifically, the articles numbered 1-5, 20-25, 27-29, 37, 40-41, and 43 in the original references were not replaced, and they were numbered 1-5, 20-25, 27-29, 49, 52-53, and 55 in the revised manuscript. In addition, in the revised paper, we added 36 new references, which are numbered 6-19, 26, 30-48, and 50-51.

**Additional Notes：**

This revision does not involve the main technical content of the research. Based on the professional opinions of the reviewers, we focused on improving the theoretical basis of the article and modified other details that can improve the readability of the research. In addition, in the revised article, all images are .tif format files modified using the PACE tool. Finally, when submitting, we will upload the images in the article and the text data used in the research in the form of a compressed file.

We appreciate the editors'/reviewers earnest work and hope that the correction will meet with approval.

Once again, thank you very much for your comments and suggestions.

Sincerely,

Corresponding author.
